# Inequities in contraceptive use among adults in Lebanon: a national study

**DOI:** 10.7189/jogh.16.04216

**Published:** 2026-07-03

**Authors:** Rindala Fayyad, Sasha Fahme, Ghada E Saad, Myriam Dagher, Hala Ghattas, Jocelyn DeJong, Stephen J McCall, WOMENA Study Group, Rindala Fayyad, Sasha Fahme, Ghada E Saad, Myriam Dagher, Hala Ghattas, Ali Abboud, Nisreen Salti, Serena Canaan, Malak Ghezzawi, Pamela Zgheib, Rita Itani, Hazar Shamas, Jocelyn DeJong, Stephen J McCall

**Affiliations:** 1 Center for Research on Population and Health, Faculty of Health Sciences, American University of Beirut, Beirut, Lebanon; 2 Department of Epidemiology and Population Health, Faculty of Health Sciences, American University of Beirut, Beirut, Lebanon; 3 Center for Global Health, Department of Medicine, Weill Cornell Medicine, New York City, USA; 4 Department of Health Promotion, Education, and Behavior, Arnold School of Public Health, University of South Carolina, Columbia, USA; 5 Faculty of Health Sciences, American University of Beirut, Beirut, Lebanon

## Abstract

**Background:**

The use of contraception is a key component of sexual and reproductive health, yet Lebanon still faces barriers that hinder the effective and equitable use of contraceptives and related reproductive health services. We aimed to determine the prevalence of contraceptive use, unmet need for family planning, demand for family planning satisfied with modern methods, and determinants of contraceptive use and modern contraceptive use among adults in Lebanon.

**Methods:**

We performed a national cross-sectional telephone survey study among adult men and women of reproductive age in Lebanon from January 2024 to July 2024. Determinants included demographic-, socioeconomic-, and health-related variables. Primary outcomes were the utilisation of any contraception method, long-acting reversible contraception methods, modern methods, and natural methods. We fitted adjusted survey-weighted logistic regression models, stratified by sex, for each determinant/outcome pair, and reported odds ratios and 95% confidence intervals as analytical outcomes.

**Results:**

We included 3146 sexually active adults of reproductive age (mean age of 38.2 years, 43.4% female, 35.8% non-Lebanese, and 14.3% with pregnancy intentions). The prevalence of contraceptive use was 58.0%, with 31.3% of respondents using modern methods, 10.3% using long-acting reversible contraceptives, and 26.7% using natural methods. In terms of sex differences, 32.7% of women and 36.4% of men had unmet need for family planning, while 37.5% and 33.9% had their demand satisfied with modern methods, respectively. Age, nationality, marital status, employment status, self-rated physical health, depression, and food insecurity were associated with contraceptive use.

**Conclusions:**

Our results offer updated national estimates of the prevalence of contraceptive use among adults in Lebanon and point to inequities in contraceptive use. There is a need to strengthen sexual and reproductive health policies, and to implement national interventions that promote the use of modern contraceptive methods for everyone in need, irrespective of age, sex, nationality, marital, or socioeconomic status.

Contraception is a key component of sexual and reproductive health (SRH) and plays a critical role in reducing maternal and infant mortality, preventing unintended pregnancies, encouraging gender equality, and socioeconomic development [1,2]. A wide range of contraception methods exist, ranging from natural ones (*e.g.* periodic abstinence, lactational amenorrhea, and withdrawal) to more effective, modern alternatives (*e.g.* hormonal contraceptives, intrauterine devices, barrier devices, emergency contraceptives, and permanent contraceptives) [3–5]. Greater access to modern contraceptives has led to significant declines in maternal deaths globally through the reduction of the number of high-risk pregnancies and unsafe abortions, as well as by enabling women to ‘space’ their pregnancies and thus avoid complications associated with short interpregnancy intervals [6–8]. Additionally, consistent condom use has significantly reduced the risk of sexually transmitted infections [9–12]. Modern contraceptive access has contributed to gender equality by granting women greater autonomy over their reproductive choices, while facilitating higher educational attainment, increased workforce participation, and improved economic stability at both national and household levels [13–15]. As such, Sustainable Development Goal target 3.7 was created within the 2030 Agenda for Sustainable Development to ‘ensure universal access to sexual and reproductive health care services, including for family planning, information and education, and the integration of reproductive health into national strategies and programmes’ [16,17].

While the Middle East and North Africa (MENA) region has witnessed progress in family planning efforts in recent years, many countries and sub-populations in the region still face significant barriers that hinder use of SRH services [18–23]. In Lebanon, contraceptive use is shaped by a complex interplay of economic factors, health care accessibility and rights, and sociopolitical dynamics, particularly among vulnerable populations [24–31]. Approximately 20% of Lebanon’s population are refugees (the majority of whom are women and children), making it the country with the highest refugee population per capita globally [32,33]. The ongoing refugee, humanitarian, and economic crises have impacted health care service delivery and the sustainability of programmes in the country, limiting governmental and non-governmental efforts to provide accessible and equitable health care for everyone [34–44]. Lebanon’s complex health system which includes both private and public providers, coupled with the severe economic crisis, has reduced availability, accessibility, and affordability of SRH services, which was found to be associated with higher rates of unintended pregnancies, unmet contraceptive needs, and maternal deaths, particularly among marginalised communities [26–31,45–56].

No national-level study in Lebanon has attempted to link determinants of health to contraceptive use across both nationals and non-nationals (including refugees). Although global and regional estimates exist, the country lacks up-to-date, nationally-representative estimates for the prevalence of contraceptive use. Furthermore, most SRH studies in the MENA region have been restricted to married women, resulting in a paucity of evidence on men and women who are sexually active outside of marriage [56–61]. These limitations in the existing evidence base have together created obstacles for data-informed interventions and SRH policies that push for universal SRH coverage. To address these knowledge gaps, we aimed to estimate the prevalence of contraceptive use, unmet need for family planning, and proportion of demand for family planning satisfied with modern methods, and identify social determinants of contraceptive use and modern contraceptive use among adults residing in Lebanon, with analyses stratified by sex.

## METHODS

This national cross-sectional study, nested within a larger parent study, aimed to identify the prevalence of and inequities in contraceptive use among women and men in Lebanon. A telephone survey was delivered across all Lebanese governorates from January 2024 to July 2024. We report our findings per the STROBE guidelines [62].

### Sampling design and study participants

The parent study aimed to identify opportunities to improve the lived experience and health of working women in the MENA region [63]. It included adults aged 19–64 years who were recruited through random digit dialling, where randomly generated numbers following the 11-digit structure of Lebanese mobile phone numbers were dialled a maximum of two times between 9:00 AM and 7:00 PM from Monday to Saturday. Calls were rescheduled if respondents expressed preference in completing the survey at a later time.

Screening questions were asked before the start of the survey to confirm respondents’ eligibility and verbal consent to participate was obtained before proceeding. The parent study included residents of Lebanon (*i.e.* temporary visitors were excluded) who were 19–64 years of age at the time of data collection. Employed women were oversampled to ensure a balanced sample in terms of sex and employment.

To estimate the prevalence of contraceptive use in this sub-study, we included all sexually active adults of reproductive age (19–49 years for women and 19–64 years for men). Survey respondents were considered sexually active if they responded that they had ever been sexually active and had an intimate partner during the previous three years.

To determine the unmet need for family planning, the proportion of demand for family planning satisfied with modern methods, and the social determinants of contraceptive use, we further restricted the sample to sexually active adults of reproductive age who stated they had no pregnancy intentions.

### Data sources and collection

We developed the survey in both English and Arabic using existing validated scales and evaluated it to ensure comprehensibility and contextual relevance. The survey for the parent study included multiple modules, with those related to sociodemographic and household characteristics, self-reported physical and mental health conditions, employment history, income, food and water security, and SRH being relevant for this study.

Trained data collectors gathered data using a computer-assisted telephone survey *via* SurveyCTO, version 2.81.2 (Dobility Inc, Cambridge, Massachusetts, USA). Data quality was monitored throughout the entire collection period, with the research team and data collectors recording and cross-checking 5% of the survey, addressing systematic errors on a weekly basis, and conducting call-backs when necessary. Data collectors gave participants the choice to abstain from the SRH section of the survey. Those who consented to participate were assigned an interviewer of the same sex and were asked to respond to questions in a private location.

### Outcomes

The primary outcomes of interest were contraceptive use, natural contraceptive use, modern contraceptive use, and long-acting reversible contraception (LARC) use, all of which were treated as categorical, binary variables. Non-modern methods were referred to as ‘natural’ methods and included lactational amenorrhea, periodic abstinence, and withdrawal. Modern methods included LARCs, in addition to female sterilisation, male sterilisation, oral contraceptive pills, male condoms, female condoms, diaphragms, and contraceptive gels. The LARC methods included intrauterine devices, contraceptive implants, and contraceptive injections [3–5]. Questions on contraceptive use referred to methods used by the respondent or their partner.

The secondary outcomes of interest were unmet need for family planning and demand for family planning satisfied with modern methods. Following United Nations Population Fund (UNFPA) definitions, with a minor difference in age range (19–49 years for women instead of 15–49 years), unmet need for family planning was defined as the percentage of individuals of reproductive age who did not have any intention to get pregnant at the time of the survey, but were not using any method of contraception [17]. Proportion of demand for family planning satisfied with modern methods was defined per the aforementioned definition as the percentage of individuals of reproductive age who did not have any intention to get pregnant and were using modern methods of contraception [17].

### Determinants

The social determinants of interest included demographic characteristics, socioeconomic status, and health-related conditions. Scales used in this study have been validated in the region [64–68].

Demographic characteristics included age, biological sex, nationality (‘Lebanese’ or ‘non-Lebanese’), and marital status (‘married/engaged’ or ‘single/divorced/widowed’). Socioeconomic variables included employment status (‘employed’ or ‘unemployed’), most recent income level (‘high income’: income equal to or above median income of participants; ‘low income’: income below median income of participants; ‘not reported’), receipt of cash assistance from humanitarian organisations (‘yes’ or ‘no’), education level (‘at least high-school degree’ or ‘less than high-school degree’), water insecurity (as defined by the Household Water Insecurity Experiences-4 (HWISE-4) scale, a brief survey tool used to measure a household’s level of water insecurity [65]: ‘water insecure’ if HWISE-4 score ≥4, and ‘water secure’ if HWISE-4 score <4), and food insecurity (as defined by the Arab Family Food Security Scale (AFFSS), a survey tool used to measure the severity of food insecurity in Arab households [66]: ‘food insecure’ if AFFSS score ≥3 and ‘food secure’ if AFFSS score <3).

Health-related variables included self-reported physical and mental health. Self-rated physical health was assessed by asking participants to rate their overall physical health as ‘poor’, ‘fair’, ‘good’, ‘very good’, or ‘excellent’. The variable was later recategorised as ‘very good/excellent’, ‘good’, and ‘poor/fair’. Anxiety was assessed through the Generalized Anxiety Disorder-7 (GAD-7) [69], a seven-item questionnaire used to screen for generalised anxiety disorder and measure the severity of anxiety symptoms, with responses grouped into three categories: none/minimal (GAD-7 score 0–4), mild (GAD-7 score 5–9), and moderate to severe (GAD-7 score 10–21). Depression was measured using the Patient Health Questionnaire-9 (PHQ-9) [70], a nine-item questionnaire used to screen for depression and measure the severity of depressive symptoms, with responses grouped into three categories: none/minimal (PHQ-9 score 0–4), mild (PHQ-9 score 5–9), and moderate to severe (PHQ-9 score 10–27). Additional health-related variables included the presence of at least one self-reported chronic medical condition (‘yes’ or ‘no’), and the presence of at least one self-reported disability (‘yes’ or ‘no’).

### Statistical analysis

We computed selection weights to account for the oversampling of employed women and post-calibration weights to ensure a representative sample in terms of nationality.

In the initial set of analyses, we generated survey-weighted descriptive statistics and prevalence, alongside their 95% confidence intervals (CIs), for each subgroup of the population to estimate the distribution of the determinant variables within each of the outcomes of interest. We then ran survey-weighted χ^2^ tests to assess the two-way interactions between each determinant and primary outcome, and to ascertain the existence of significant variations in the reported contraceptive methods used among different subgroups of the population. We also investigated and reported on specific methods of contraception used or reasons for not using contraception.

For the adjusted analyses, we fitted separate multivariable survey-weighted logistic regression models for each determinant/primary outcome pair, while adjusting for potential confounders (Table S1 in the **Online Supplementary Document**). Confounders were identified by constructing directed acyclic graphs for each determinant and outcome. These graphs were developed based on literature reviews and were agreed upon by team members after discussing all pathways and underlying assumptions. We reported on our assessment of the association between each determinant and primary outcome using adjusted odds ratios (aORs) and 95% CIs. All analyses were conducted separately for men and women.

Given that less than 2% of the population had missing data, we conducted a complete-case analysis. We performed all analyses in *R*, version 4.4.2 (R Core Team, Vienna, Austria). A *P*-value <0.05 indicated statistical significance.

## RESULTS

**Figure 1:**
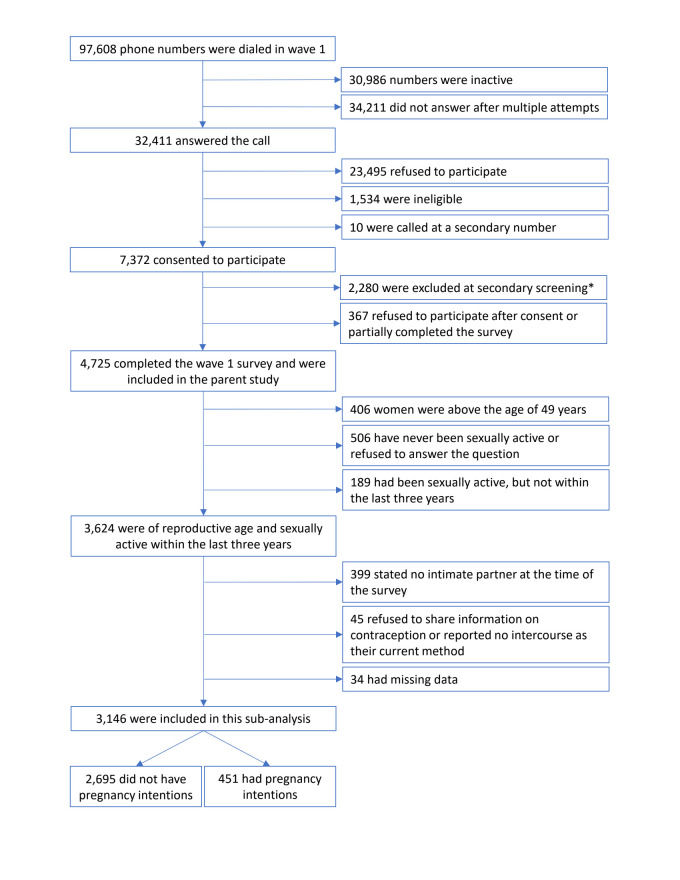
Flow diagram for the study sample selection. *At this stage, we further assessed participants’ employment status and sex to enable oversampling of employed women to align with national estimates.

The parent study reached out to 97 608 phone numbers; 32 411 individuals responded and 7372 were eligible and consented to participate. Our current sub-analysis enrolled 3146 participants, including 451 individuals who had pregnancy intentions (**Figure 1**). The mean age of this sample was 38.2 years (standard deviation = 10.1), with 43.4% of participants being female, 35.8% being non-Lebanese, and 41.3% being unemployed (Table S2 in the **Online Supplementary Document**). Most non-Lebanese individuals surveyed were Syrian (92.9%) and Palestinian (5.2%) refugees or migrants (Table S3 in the **Online Supplementary Document**).

In this sample, 60.1% (95% CI = 57.3–63.0) of women and 56.3% (95% CI = 54.0–58.6) of men reported that they or their partner used some method of contraception (**Table 1**; Table S4 in the **Online Supplementary Document**). Approximately a third of the female and male populations who were not trying to get pregnant had unmet need for family planning, and approximately a third had their demand satisfied with modern methods (**Table 2**; Table S5 in the **Online Supplementary Document**). Unmet need for family planning and demand satisfied with modern methods varied by marital status. Among single, widowed, or divorced women, 93.5% (95% CI = 89.1–97.9) had unmet need for family planning and only 4.9% (95% CI = 1.1–8.6) had their demand satisfied with modern methods, compared to 23.8% (95% CI = 20.9–26.7) and 42.3% (95% CI = 39.0–45.6), respectively, among married or engaged women (**Table 2**). We found a similar pattern by marital status among men (Table S5 in the **Online Supplementary Document**). Non-Lebanese women and men were significantly more likely to have their need for family planning met and to have their demand for family planning satisfied with modern methods, compared to Lebanese citizens (**Table 2**; Table S5 in the **Online Supplementary Document**).

**Table 1:** Weighted prevalence estimates (in %) of contraceptive use and modern contraceptive use among women aged 19–49 years or their partner in Lebanon in 2024, overall and stratified by social determinant*

	Prevalence of contraceptive use (95% CI)			Prevalence of types of contraception among users only (95% CI)				
	**No**	**Yes**	**Denominator (weighted/unweighted)**	**Modern**	**Natural**	**LARC**	**Non-LARC**	**Denominator (weighted/unweighted)**
**Total**	39.9 (37.0–42.7)	60.1 (57.3–63.0)	2099/1293	54.8 (51.1–58.5)	45.2 (41.5–48.9)	18.2 (15.3–21.0)	81.8 (79.0–84.7)	1262/776
**Age in years**								
19–29	51.9 (46.2–57.5)	48.1 (42.5–53.8)	551/342	56.0 (47.8–64.3)	44.0 (35.7–52.2)	19.8 (13.3–26.4)	80.2 (73.6–86.7)	266/159
30–39	34.0 (29.7–38.2)	66.0 (61.8–70.3)	883/550	53.3 (47.8–58.8)	46.7 (41.2–52.2)	18.0 (14.0–22.1)	82.0 (77.9–86.0)	583/363
40–49	37.8 (32.7–42.9)	62.2 (57.1–67.3)	666/401	56.1 (49.6–62.6)	43.9 (37.4–50.4)	17.2 (12.3–22.1)	82.8 (77.9–87.7)	414/254
*P*-value		<0.001			0.771		0.811	
**Nationality**								
Lebanese	41.8 (38.2–45.5)	58.2 (54.5–61.8)	1423/790	51.7 (46.9–56.6)	48.3 (43.4–53.1)	15.6 (12.1–19.1)	84.4 (80.9–87.9)	828/456
Non-Lebanese	35.8 (31.4–40.2)	64.2 (59.8–68.6)	676/503	60.6 (55.0–66.2)	39.4 (33.8–45.0)	23.0 (18.2–27.8)	77.0 (72.2–81.8)	434/320
*P*-value		0.039			0.020		0.013	
**Education level**								
High-school degree or higher	39.2 (35.1–43.2)	60.8 (56.8–64.9)	1001/628	51.5 (46.2–56.9)	48.5 (43.1–53.8)	16.2 (12.3–20.0)	83.8 (80.0–87.7)	609/377
Less than high-school degree	40.5 (36.5–44.5)	59.5 (55.5–63.5)	1098/665	57.8 (52.7–63.0)	42.2 (37.0–47.3)	20.0 (15.9–24.1)	80.0 (75.9–84.1)	653/399
*P*-value		0.644			0.098		0.181	
**Marital status**								
Married/engaged	33.2 (30.3–36.1)	66.8 (63.9–69.7)	1867/1141	54.6 (50.8–58.3)	45.4 (41.7–49.2)	18.4 (15.5–21.2)	81.6 (78.8–84.5)	1247/767
Single/divorced/widowed	93.5 (89.2–97.9)	6.5 (2.1–10.8)	232/152	74.9 (43.7–106.2)	25.1 (0.0–56.3)	0.0 (0.0–0.0)	100.0 (100.0–100.0)	15/9
*P*-value		<0.001			0.269		0.179	
**Employment status**								
Employed	42.3 (38.4–46.1)	57.7 (53.9–61.6)	729/654	54.4 (49.3–59.5)	45.6 (40.5–50.7)	19.8 (15.8–23.9)	80.2 (76.1–84.2)	421/380
Unemployed	38.6 (34.8–42.5)	61.4 (57.5–65.2)	1370/639	55.0 (50.0–60.0)	45.0 (40.0–50.0)	17.3 (13.6–21.0)	82.7 (79.0–86.4)	842/396
*P*-value		0.188			0.873		0.364	
**Income level**								
High income (≥median)	42.3 (36.4–48.1)	57.7 (51.9–63.6)	431/310	54.8 (47.0–62.5)	45.2 (37.5–53.0)	17.4 (11.7–23.2)	82.6 (76.8–88.3)	249/179
Low income (<median)	40.4 (35.0–45.7)	59.6 (54.3–65.0)	501/382	55.1 (48.2–61.9)	44.9 (38.1–51.8)	17.5 (12.5–22.5)	82.5 (77.5–87.5)	298/233
Not reported	38.8 (34.7–42.8)	61.2 (57.2–65.3)	1167/601	54.7 (49.4–60.0)	45.3 (40.0–50.6)	18.7 (14.6–22.8)	81.3 (77.2–85.4)	714/364
*P*-value		0.611			0.996		0.901	
**Received cash assistance**								
No	41.7 (38.5–44.9)	58.3 (55.1–61.5)	1671/1005	52.8 (48.4–57.1)	47.2 (42.9–51.6)	16.3 (13.1–19.4)	83.7 (80.6–86.9)	974/579
Yes	32.8 (26.9–38.6)	67.2 (61.4–73.1)	429/288	61.7 (54.4–69.0)	38.3 (31.0–45.6)	24.5 (18.2–30.8)	75.5 (69.2–81.8)	289/197
*P*-value		0.011			0.044		0.014	
**Self-rated physical health**								
Very good/excellent	54.6 (45.4–63.8)	45.4 (36.2–54.6)	201/128	53.3 (39.8–66.7)	46.7 (33.3–60.2)	21.6 (10.1–33.1)	78.4 (66.9–89.9)	92/60
Good	42.5 (37.8–47.3)	57.5 (52.7–62.2)	772/469	55.8 (49.4–62.1)	44.2 (37.9–50.6)	17.1 (12.5–21.8)	82.9 (78.2–87.5)	444/268
Poor/fair	35.4 (31.6–39.2)	64.6 (60.8–68.4)	1126/696	54.4 (49.5–59.3)	45.6 (40.7–50.5)	18.3 (14.6–22.0)	81.7 (78.0–85.4)	727/448
*P*-value		<0.001			0.921		0.749	
**Anxiety severity**								
Minimal	45.5 (40.2–50.8)	54.5 (49.2–59.8)	602/377	52.2 (44.9–59.5)	47.8 (40.5–55.1)	20.5 (14.7–26.3)	79.5 (73.7–85.3)	328/204
Mild	39.4 (34.7–44.2)	60.6 (55.8–65.3)	741/455	51.1 (44.8–57.5)	48.9 (42.5–55.2)	15.9 (11.4–20.4)	84.1 (79.6–88.6)	448/273
Moderate to severe	35.8 (31.1–40.5)	64.2 (59.5–68.9)	756/461	59.9 (54.0–65.9)	40.1 (34.1–46.0)	18.6 (14.0–23.2)	81.4 (76.8–86.0)	485/299
*P*-value		0.029			0.102		0.451	
**Depression severity**								
None to minimal	44.8 (38.4–51.3)	55.2 (48.7–61.6)	413/256	52.4 (43.6–61.2)	47.6 (38.8–56.4)	16.3 (10.0–22.7)	83.7 (77.3–90.0)	228/139
Mild	43.0 (38.1–47.8)	57.0 (52.2–61.9)	728/455	55.8 (49.4–62.3)	44.2 (37.7–50.6)	21.1 (15.9–26.4)	78.9 (73.6–84.1)	416/260
Moderate to severe	35.4 (31.2–39.6)	64.6 (60.4–68.8)	958/582	55.0 (49.6–60.4)	45.0 (39.6–50.4)	16.8 (12.9–20.7)	83.2 (79.3–87.1)	619/377
*P*-value		0.016			0.824		0.347	
**Any chronic medical condition**								
No	40.3 (36.7–43.8)	59.7 (56.2–63.3)	1350/838	51.8 (47.2–56.5)	48.2 (43.5–52.8)	17.7 (14.2–21.1)	82.3 (78.9–85.8)	806/498
Yes	39.2 (34.4–44.0)	60.8 (56.0–65.6)	750/455	60.0 (53.8–66.2)	40.0 (33.8–46.2)	19.0 (14.1–23.9)	81.0 (76.1–85.9)	456/278
*P*-value		0.724			0.041		0.652	
**Any disability**								
No	40.2 (37.3–43.1)	59.8 (56.9–62.7)	2018/1240	55.2 (51.3–59.0)	44.8 (41.0–48.7)	17.8 (14.9–20.7)	82.2 (79.3–85.1)	1207/739
Yes	32.4 (18.7–46.0)	67.6 (54.0–81.3)	82/53	46.9 (29.6–64.2)	53.1 (35.8–70.4)	25.8 (11.0–40.5)	74.2 (59.5–89.0)	55/37
*P*-value		0.294			0.359		0.243	
**Water insecure**								
No	41.4 (37.3–45.4)	58.6 (54.6–62.7)	1051/650	55.0 (49.6–60.3)	45.0 (39.7–50.4)	19.9 (15.6–24.2)	80.1 (75.8–84.4)	616/380
Yes	38.4 (34.4–42.4)	61.6 (57.6–65.6)	1049/643	54.6 (49.4–59.9)	45.4 (40.1–50.6)	16.5 (12.8–20.2)	83.5 (79.8–87.2)	646/396
*P*-value		0.306			0.928		0.245	
**Food insecur**e								
No	47.9 (40.9–55.0)	52.1 (45.0–59.1)	334/218	54.1 (44.5–63.7)	45.9 (36.3–55.5)	23.8 (15.5–32.1)	76.2 (67.9–84.5)	174/114
Yes	38.4 (35.2–41.5)	61.6 (58.5–64.8)	1765/1075	54.9 (50.8–59.0)	45.1 (41.0–49.2)	17.3 (14.3–20.2)	82.7 (79.8–85.7)	1088/662
*P*-value		0.013			0.878		0.115	

CI – confidence interval, LARC – long-acting reversible contraception

*Reported *P*-values were extracted from χ^2^ tests, assessing contraceptive use differences among subgroups of the population.

**Table 2:** Weighted prevalence estimates (in %) of unmet need for family planning and demand satisfied with modern methods among women aged 19–49 years in Lebanon in 2024, overall and stratified by social determinant

	Prevalence of unmet need for family planning (95% CI)*	Percentage of demand satisfied with modern methods (95% CI)*	Denominator (weighted/unweighted)
**Total**	32.7 (29.7–35.6)	37.5 (34.5–40.5)	1812/1119
**Age in years**			
19–29	43.2 (37.1–49.4)	32.4 (26.6–38.3)	453/281
30–39	25.3 (21.1–29.5)	40.5 (35.8–45.3)	746/467
40–49	33.8 (28.7–39.0)	37.6 (32.3–42.9)	613/371
**Nationality**			
Lebanese	35.2 (31.4–38.9)	34.4 (30.6–38.2)	1239/692
Non-Lebanese	27.3 (22.9–31.7)	44.2 (39.3–49.2)	573/427
**Education level**			
High-school degree or higher	33.0 (28.8–37.1)	35.2 (31.0–39.5)	883/553
Less than high-school degree	32.4 (28.3–36.6)	39.7 (35.4–44.0)	929/566
**Marital status**			
Married/engaged	23.8 (20.9–26.7)	42.3 (39.0–45.6)	1580/968
Single/divorced/widowed	93.5 (89.1–97.9)	4.9 (1.1–8.6)	232/151
**Employment status**			
Employed	35.8 (31.8–39.7)	35.1 (31.2–39.1)	642/574
Unemployed	31.0 (27.0–35.0)	38.8 (34.7–43.0)	1170/545
**Income level**			
High income (≥median)	36.1 (30.1–42.1)	36.0 (30.0–41.9)	375/273
Low income (<median)	33.1 (27.5–38.6)	37.7 (32.0–43.3)	431/328
Not reported	31.2 (27.1–35.4)	38.0 (33.7–42.4)	1006/518
**Received cash assistance**			
No	34.1 (30.7–37.4)	35.4 (31.9–38.8)	1429/862
Yes	27.5 (21.6–33.4)	45.6 (39.1–52.1)	383/257
**Self-rated physical health**			
Very good/excellent	42.8 (32.6–53.0)	30.5 (20.8–40.1)	160/103
Good	36.2 (31.2–41.2)	36.6 (31.6–41.6)	666/405
Poor/fair	28.6 (24.8–32.5)	39.3 (35.1–43.4)	986/611
**Anxiety severity**			
Minimal	37.7 (32.1–43.3)	33.0 (27.5–38.5)	511/321
Mild	32.3 (27.3–37.2)	35.8 (30.7–40.8)	630/388
Moderate to severe	29.2 (24.5–34.0)	42.6 (37.5–47.7)	671/410
**Depression severity**			
None to minimal	35.4 (28.6–42.2)	34.1 (27.2–41.0)	340/209
Mild	35.8 (30.7–40.8)	36.7 (31.6–41.8)	626/397
Moderate to severe	29.3 (25.1–33.5)	39.5 (35.0–44.0)	846/513
**Any chronic medical condition**			
No	32.4 (28.7–36.1)	35.8 (32.0–39.5)	1150/715
Yes	33.1 (28.2–38.0)	40.5 (35.4–45.6)	662/404
**Any disability**			
No	32.9 (29.9–35.9)	37.5 (34.4–40.6)	1742/1073
Yes	25.9 (12.0–39.9)	37.3 (22.6–52.0)	70/46
**Water insecure**			
No	35.1 (30.9–39.3)	36.7 (32.5–41.0)	906/562
Yes	30.3 (26.2–34.3)	38.3 (34.0–42.6)	906/557
**Food insecure**			
No	40.4 (32.8–47.9)	33.5 (26.3–40.8)	280/183
Yes	31.3 (28.1–34.4)	38.2 (34.9–41.6)	1532/936

CI – confidence interval

*Definitions of the indicators follow those provided by the United Nations Population Fund State of the World Population Report [17].

**Figure 2:**
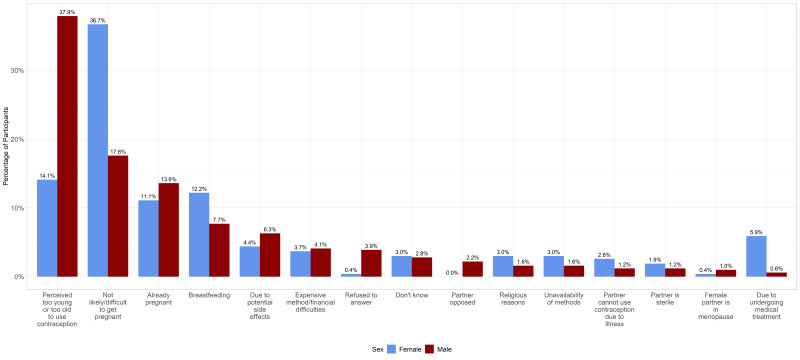
Weighted distribution of reasons for not using contraception among individuals of reproductive age with no pregnancy intentions, stratified by sex.

The intrauterine device was the most frequently used LARC method by participants or their partner with no pregnancy intentions, reported by 11.9% of women and 10.8% of men (**Table 3**; Table S6 in the **Online Supplementary Document**). Overall, LARC methods were used almost exclusively by married or engaged couples. The modern non-LARC method used most frequently was the oral contraceptive pill, and the natural method used most frequently was withdrawal (**Table 3**; Table S6 in the **Online Supplementary Document**). Women stated that the main reason they or their partner did not use contraception was because it was unlikely or difficult for them to get pregnant (36.7%), while the main reason stated by men was that they perceived themselves or their partner to be too young or too old to use contraception (37.9%) (**Figure 2**).

**Table 3:** Distribution of contraceptive methods reported by women aged 19–49 years with no pregnancy intentions in Lebanon in 2024, including methods used by themselves or their partners, stratified by marital status*

Method of contraception	Total (n = 1119)	Married/engaged (n = 968)	Single/divorced/widowed (n = 151)
**Nothing**	367 (32.7)	225 (23.8)	142 (93.5)
**Natural methods**			
*Withdrawal*†	309 (27.9)	307 (31.8)	2 (1.7)
*Periodic abstinence*†	43 (3.8)	42 (4.1)	1 (1.3)
*LAM*‡	6 (0.6)	6 (0.7)	0 (0.0)
**Modern methods**			
LARC methods			
*Intrauterine device*‡	139 (11.9)	139 (13.6)	0 (0.0)
*Contraceptive injection*‡	4 (0.3)	4 (0.3)	0 (0.0)
*Contraceptive implant*‡	3 (0.2)	3 (0.3)	0 (0.0)
Non-LARC modern methods			
*Oral contraceptive pill‡*	159 (14.1)	158 (16.1)	1 (0.4)
*Male condom§*	84 (7.7)	79 (8.2)	5 (3.9)
*Female sterilisation‡*	20 (2.1)	19 (2.3)	1 (0.4)
*Female condom‡*	6 (0.6)	6 (0.6)	0 (0.0)
*Diaphragm‡*	9 (0.7)	9 (0.8)	0 (0.0)
*Male sterilisation§*	2 (0.2)	2 (0.3)	0 (0.0)
*Contraceptive gels‡*	0 (0.0)	0 (0.0)	0 (0.0)

LAM – lactational amenorrhea method, LARC – long-acting reversible contraception

*Presented as n (weighted %).

†Applicable to males and females.

‡Female-specific.

§Male-specific.

**Figure 3:**
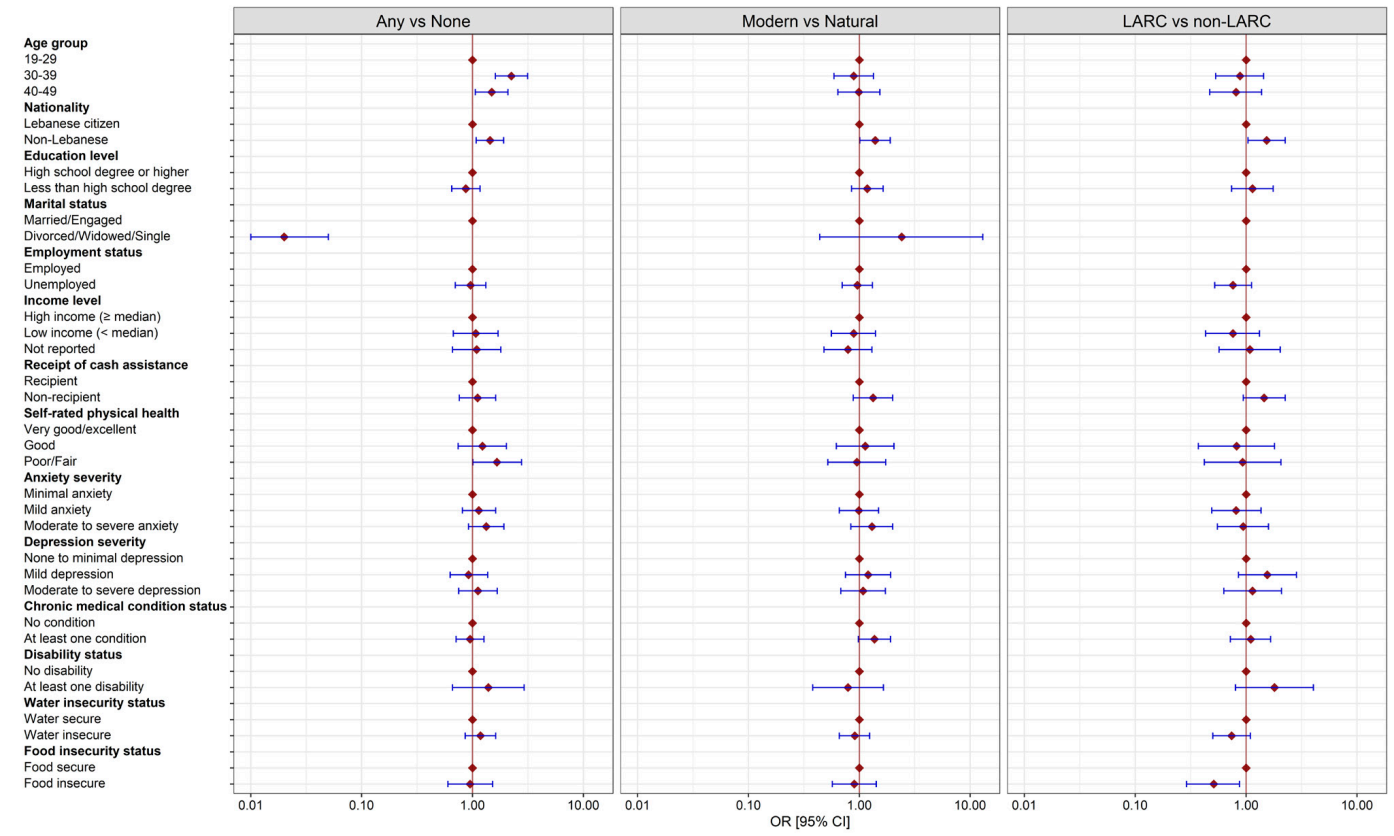
Forest plot for the adjusted odds ratio estimates and 95% confidence intervals from survey-weighted logistic regression models for the effects of each determinant on method of contraception used among women with no pregnancy intentions. Confounders for each model can be found in Table S1 in the **Online Supplementary Document****.** Outcome coding: any = 1, none = 0; modern = 1, natural = 0; LARC = 1, non-LARC = 0. CI – confidence interval, OR – odds ratio.

In terms of adjusted estimates for the relationships of interest among women with no pregnancy intentions, we identified inequities in contraceptive use by age, nationality, marital status, and self-rated physical health (**Figure 3**; Table S7 in the **Online Supplementary Document**). Specifically, women with significantly higher odds of using any method of contraception included those aged 30–39 years (aOR = 2.24; 95% CI = 1.61–3.14) and those aged 40–49 years (aOR = 1.49; 95% CI = 1.06–2.09) compared to women aged 19–29 years; non-nationals compared to nationals (aOR = 1.44; 95% CI = 1.08–1.91); married or engaged women compared to single, divorced, or widowed women (aOR = 50.00; 95% CI = 20.00–100.00); and women with poor to fair self-rated physical health compared to those who rated their health as very good or excellent (aOR = 1.66; 95% CI = 1.01–2.77).

We observed inequities in modern contraceptive use by nationality among women using at least one method of contraception, and inequities in LARC use by nationality and food insecurity status (**Figure 3**; Table S7 in the **Online Supplementary Document**). Non-Lebanese women had 1.39 (95% CI = 1.01–1.90) and 1.53 (95% CI = 1.04–2.25) times higher odds of using modern methods and LARCs respectively, compared to Lebanese women. Women from food secure households had 1.96 (95% CI = 1.15–3.45) times higher odds of using LARCs compared to those with food insecurity.

Concerning the adjusted estimated associations among men with no pregnancy intentions, we identified inequities in contraceptive use by age, nationality, and employment status; inequities in modern contraceptive use by nationality and depression severity; and inequities in LARC use by nationality (Figure S1 and Table S8 in the **Online Supplementary Document**). Specifically, non-Lebanese men had 1.30 (95% CI = 1.04–1.64) times higher odds of using any contraceptive method; among those reporting contraceptive use, they had 1.73 (95% CI = 1.34–2.25) and 1.49 (95% CI = 1.07–2.08) times higher odds of using modern methods and LARCs, respectively, compared to Lebanese men. Additionally, employed men had 1.45 (95% CI = 1.09–1.92) times higher odds of using any contraceptive compared to those unemployed, and men with mild depression had 1.56 (95% CI = 1.11–2.21) times higher odds of using modern methods compared to those with no to minimal depression.

## DISCUSSION

Our results demonstrate that, among a large sample of sexually active reproductive-age adults, over half of Lebanese women, Lebanese men, and non-Lebanese men, and nearly two thirds of non-Lebanese women reported using contraceptives. Among women and men with no pregnancy intentions, approximately one third had unmet need for family planning, and over one third had their demand for family planning satisfied with modern methods. We also identified age, nationality, marital status, employment status, self-rated physical health, depression, and food insecurity to be associated with contraceptive use.

Less than 30% of sampled Lebanese citizens of reproductive age (aged 19 years and above) reported modern contraceptive use – a figure that is below the 2024 regional average of 35.4% (which includes a broader age group starting at 15 years old) [71]. Contraceptive use in humanitarian and crisis settings is often compromised due to a combination of disrupted health systems, damaged infrastructure, broken supply chains, and de-prioritisation of SRH and family planning services during emergencies, despite its inclusion as a key objective in the Minimum Initial Service Package for reproductive health [16,72]. Global evidence has demonstrated persistent and widespread inequities in contraceptive use across many conflict-impacted and low-resource settings, with disparities driven by socioeconomic, demographic, sociocultural, and geographic factors [73–94].

Overlapping and protracted economic, political, and security crises have strained Lebanon’s health care system, shifting the focus from creating sustainable, long-term interventions to placing heavier emphasis on emergency and acute care [34–44]. While family planning services are available in the private sector, their cost has made them inaccessible for a large proportion of the population, increasing calls for support from non-governmental organisations and humanitarian actors [26–29,51–54]. However, healthcare service provision from these actors is often limited and unevenly distributed, making healthcare access and modern contraceptive use reliant on an individual’s ability to navigate the health system, rather than being solely determined by need or demand [52,95–97]. In such contexts, the low prevalence of modern contraceptive use may reflect structural difficulties in sustaining inclusive and comprehensive SRH and family planning services during crises, rather than a simple result of individual-level barriers.

Observed differences in contraceptive use based on nationality may be attributable to differential health care seeking behaviour, as many Lebanese nationals prefer to access care through the private sector, where free or low-cost SRH services are not offered. The private sector has largely dominated Lebanon’s health care system for decades, making most services highly specialised and expensive [34,98–101]. Although primary health care centres exist and provide preventative care services for all individuals, irrespective of citizenship (including free or highly subsidized family planning and SRH services), refugees and migrants have constituted the majority of beneficiaries seeking care at these centres, particularly since the start of the Syrian civil war in 2011. This is likely because these centres were originally established with the needs of vulnerable populations in mind and continue to primarily serve low-income groups. Despite SRH services in primary health care centres being available to all, the number of Lebanese nationals or individuals of higher socioeconomic status that frequent these facilities remains very low [102–109]. These differences in health care seeking behaviour may explain the protective associations for non-Lebanese adults.

We found that employed men and food secure women were more likely to be on contraceptives, indicating that cost and affordability may have been major factors influencing contraceptive use. We also found that women who rated their physical health as poor or fair had higher odds of using contraception compared to those with better self-rated health, and that men with mild depression had higher odds of using modern contraception compared to those without depression. These patterns may be reflective of higher contact with the health care system among individuals managing physical or mental health concerns, providing them with more opportunities to access or be exposed to SRH promotion, services, and counselling.

We also determined that single individuals or separated couples were among the least likely to use contraception, raising important questions about the unmet need and accessibility of SRH services for unmarried individuals in Lebanon. This may be due to the stigma surrounding SRH in the MENA region, particularly as it relates to single women. In such contexts, it is not the absence of sexual activity, but rather the fear of judgment or social consequences that deters unmarried individuals from purchasing or requesting contraceptives, leading to unprotected sex and heightened risk of unintended pregnancies, unsafe abortions, and sexually transmitted infections [22,59,110]. Moreover, multiple factors may influence reason and choice of contraception in Lebanon, including limited SRH education, stigma, lack of agency for women, and documented side effects of specific contraceptive methods [3,4,26].

The main strength of this study lies in its design and setting. The most recent study in Lebanon estimating the prevalence of contraceptive use on a national scale was conducted in 2009, before the Syrian civil war and subsequent national demographic changes [71,111]. However, that study focused solely on married Lebanese women. To our knowledge, no such study has been conducted since, making this the first national study in 16 years to provide updated estimates of contraceptive use throughout the country. This is also, to our knowledge, the first study in Lebanon to investigate social determinants of contraceptive use and modern contraceptive use on a national scale, among males and females of both host and migrant/refugee populations. Furthermore, due to prevailing social and cultural sensitivities in the MENA region around single women’s sexual health, many countries cannot adequately report on the Sustainable Development Goal target 3.7 [16,17], as that target uses a denominator of all women, and not married women only. Our study, however, interviewed participants regardless of marital status, ensuring that the experiences and needs of non-married women were also captured – an approach that was made possible through the telephone survey design.

This study also has some limitations. Due to the self-reported nature of the data, there is a possibility of recall bias and data misclassification. Moreover, due to the stigma surrounding SRH, some single women may not have answered openly to all questions for fear of disclosing sexual activity outside of marriage [110]. However, participants were given the choice to opt out of the SRH section if they preferred, and those who consented to be included were assigned an interviewer of the same sex and were asked to respond to the questions in a private location (response rate of 99.6%). While we adopted the UNFPA’s definition for unmet need for family planning and demand for family planning satisfied with modern methods [17], these metrics failed to account for individuals who voluntarily chose not to use contraceptives or who were infecund and, therefore, did not have a demand for or need of contraceptives. As such, the adopted indicators are use-based rather than satisfaction- or needs-based. Moreover, due to the design of the parent study, we only included women above the age of 18 years, while the UNFPA’s definition includes women from the age of 15 years and above, which may limit its comparability. Similarly, due to the parent study’s focus on employed adults, we did not capture adult men older than 64 years. Additionally, since this study was cross-sectional in design, meaning it lacked a temporal dimension, we could not establish the causality and directionality of the observed relationships between the determinants and outcomes. This is particularly important for the association between depression and contraception use, as previous studies have shown that hormonal contraception may cause depression [112]. As with most observational studies, we also acknowledge that there may be unmeasured or residual confounding that was not accounted for. Finally, due to the telephone-based nature of the study, we did not capture individuals with no cell phones or active SIM cards. However, cell phone ownership in Lebanon is high, particularly among Syrian refugees [113].

Our findings signal a need for a response on a national level to reinforce SRH policies, promote the use of modern methods of contraception, and strengthen primary care. Interventions to reorient the highly privatised health care system towards a more people-centred primary and preventative care system that incorporates family planning are necessary to improve the usability, accessibility, and availability of health services [114]. It is also important to include sexuality education in schools, raise awareness and educate the public about where and how to access modern contraceptives, family planning, and SRH services, and train health care providers at the primary level on family planning and counselling, as well as on the administration of LARCs and other modern contraceptives. Such efforts are essential to meeting universal SRH needs in Lebanon, especially among non-married sexually active women who have not received programmatic or policy attention.

## CONCLUSIONS

In this national-level study, we estimated the prevalence of contraceptive use, unmet need for family planning, and demand for family planning satisfied with modern methods among adults in Lebanon. We also identified social factors that are significantly associated with contraceptive use throughout the country, such as age, nationality, marital status, employment status, depression, self-rated physical health, and food insecurity. Actions to strengthen the primary health care system and increase awareness about SRH and family planning services, as well as more research and surveys on SRH issues in Lebanon, are necessary to meet universal reproductive health needs.

## Additional material


Online Supplementary Document
