# Supplementary material for: Inequities in contraceptive use among adults in Lebanon: a national study
Source: J Glob Health. 2026 Jul 3;16:04216. doi: 10.7189/jogh.16.04216 (PMC13329941; doi:10.7189/jogh.16.04216)
Supplement: Online Supplementary Document [file jogh-16-04216-s001.pdf]

Supplement to: Fayyad R, Fahme S, Saad GE, Dagher M, Ghattas H, DeJong J, McCall SJ; WOMENA Study Group. Inequities in contraceptive use among adults in Lebanon: a national study. J Glob Health. 2026;16:04216.

Table of contents

1 **Table S1:** Variables confounding the relationship between each determinant and contraceptive use

2 **Table S2:** Characteristics of the overall study population

3 **Table S3:** Distribution of the different nationalities among the non-Lebanese study population

4 **Table S4:** Weighted prevalence estimates (in %) of contraceptive use and modern contraceptive use among men aged 19-64 years or their partner in Lebanon in 2024, overall and stratified by social determinant

5 **Table S5:** Weighted prevalence estimates (in %) of unmet need for family planning and demand satisfied with modern methods among men aged 19-64 years in Lebanon in 2024, overall and stratified by social determinant

6 **Table S6:** Distribution of contraceptive methods reported by men aged 19-64 years with no pregnancy intentions in Lebanon in 2024, including methods used by themselves or their partners, stratified by marital status

7 **Table S7:** Adjusted odds ratio estimates and 95% confidence intervals from survey-weighted logistic regression models for the effects of each determinant on method of contraception used among women aged 19-49 years with no pregnancy intentions

8 **Table S8:** Adjusted odds ratio estimates and 95% confidence intervals from survey-weighted logistic regression models for the effects of each determinant on method of contraception used among men aged 19-64 years with no pregnancy intentions

9 **Figure S1:** Forest plot for the adjusted odds ratio estimates and 95% confidence intervals from survey-weighted logistic regression models for the effects of each determinant on method of contraception used among men with no pregnancy intentions

Table S1: Variables confounding the relationship between each determinant and contraceptive use

	**Confounder***										
**Social determinant**	Age	Nationality	Education level	Employment status	Income	Marital status	Receipt of aid	Chronic medical condition status	Disability status	Food insecurity	Water insecurity
Age											
Nationality	X										
Education level	X	X									
Employment status	X	X	X			X		X	X		
Income level	X	X	X	X		X	X				
Marital status	X										
Receipt of aid	X	X									
Self-rated physical health	X							X	X	X	X
Anxiety severity	X	X			X		X	X	X	X	X
Depression severity	X	X			X		X	X	X	X	X
Chronic medical condition status	X										
Disability status	X										
Food insecurity	X	X			X	X	X				
Water insecurity	X	X			X	X	X				

*Some confounders may not have been captured

Table S2: Characteristics of the overall study population

Population group	n (weighted %)
**Total**	3146 (100%)
**Methods of contraception used by the participant or their partner**	
LARC method	337 (10.3%)
Non-LARC modern method	668 (21.0%)
Natural method	820 (26.7%)
Nothing	1321 (42.0%)
**Age in years**	
Mean age (SD)	38.2 (10.1)
19-29	687 (21.3%)
30-39	1126 (35.3%)
40-49	916 (29.5%)
50-59	315 (10.3%)
60-64	102 (3.5%)
**Sex**	
Female	1293 (43.4%)
Male	1853 (56.6%)
**Nationality**	
Lebanese	1747 (64.2%)
Non-Lebanese	1399 (35.8%)
**Education level**	
High-school degree or higher	1267 (41.8%)
Less than high-school degree	1879 (58.3%)
**Marital status**	
Married/engaged	2804 (89.1%)
Single/divorced/widowed	342 (10.9%)
**Pregnancy intentions**	
No	2695 (85.7%)
Yes	451 (14.3%)
**Employment status**	
Employed	2059 (58.7%)
Unemployed	1087 (41.3%)
**Income level**	
High income (≥ median)	1269 (38.9%)
Low income (< median)	975 (27.8%)
Not reported	902 (33.3%)
**Received cash assistance from humanitarian organizations**	
No	2456 (80.7%)
Yes	690 (19.3%)

**Self-rated physical health**	
Very good/excellent	476 (15.2%)
Good	1196 (38.3%)
Poor/fair	1474 (46.5%)
**Anxiety severity**	
Minimal	1161 (37.2%)
Mild	1006 (31.8%)
Moderate to severe	979 (31.0%)
**Depression severity**	
None to minimal	780 (25.3%)
Mild	1060 (33.7%)
Moderate to severe	1306 (41.0%)
**Any chronic medical condition**	
No	2086 (65.8%)
Yes	1060 (34.2%)
**Any disability**	
No	2958 (94.5%)
Yes	188 (5.5%)
**Water insecure**	
No	1731 (55.4%)
Yes	1415 (44.6%)
**Food insecure**	
No	568 (18.8%)
Yes	2578 (81.2%)

LARC – long-acting reversible contraception, SD – standard deviation

Table S3: Distribution of the different nationalities among the non-Lebanese study population

Country of nationality	n (weighted %)
Total (non-Lebanese)	n = 1399
Syria	1297 (92.9%)
Palestine	74 (5.2%)
Sudan	7 (0.5%)
Egypt	6 (0.4%)
Ethiopia	5 (0.3%)
Iraq	3 (0.2%)
Turkey	3 (0.2%)
France	1 (0.1%)
Italy	1 (0.1%)
Tunisia	1 (0.1%)
Stateless	1 (0.1%)

Table S4: Weighted prevalence estimates (in %) of contraceptive use and modern contraceptive use among men aged 19-64 years or their partner in Lebanon in 2024, overall and stratified by social determinant*

	Prevalence of contraceptive use (95% CI)			Prevalence of types of contraception among users only (95% CI)				
	**No**	**Yes**	**Denominator (weighted/ unweighted)**	**Modern**	**Natural**	**LARC**	**non-LARC**	**Denominator (weighted/ unweighted)**
**Total**	43.7 (41.4-46.0)	56.3 (54.0-58.6)	2737/1853	53.3 (50.2-56.3)	46.7 (43.7-49.8)	17.3 (15.0-19.6)	82.7 (80.4-85.0)	1542/1049
**Age in years**								
19–29	63.7 (58.5-68.8)	36.3 (31.2-41.5)	481/345	61.0 (52.3-69.6)	39.0 (30.4-47.7)	23.2 (15.8-30.7)	76.8 (69.3-84.2)	175/128
30–39	41.7 (37.6-45.8)	58.3 (54.2-62.4)	827/576	54.8 (49.4-60.2)	45.2 (39.8-50.6)	16.2 (12.3-20.2)	83.8 (79.8-87.7)	482/341
40–49	27.8 (23.9-31.7)	72.2 (68.3-76.1)	762/515	50.7 (45.5-55.9)	49.3 (44.1-54.5)	17.3 (13.4-21.2)	82.7 (78.8-86.6)	549/368
50–59	42.7 (37.2-48.2)	57.3 (51.8-62.8)	499/315	54.1 (46.8-61.5)	45.9 (38.5-53.2)	17.0 (11.5-22.5)	83.0 (77.5-88.5)	286/182
60–64	70.6 (61.7-79.5)	29.4 (20.5-38.3)	169/102	35.3 (18.1-52.4)	64.7 (47.6-81.9)	9.4 (0.0-19.8)	90.6 (80.2-100.9)	50/30
P-value		< 0.001			0.088		0.344	
**Nationality**								
Lebanese	44.8 (41.7-48.0)	55.2 (52.0-58.3)	1682/957	47.4 (43.1-51.6)	52.6 (48.4-56.9)	15.0 (11.9-18.0)	85.0 (82.0-88.1)	928/528
Non-Lebanese	41.8 (38.6-45.1)	58.2 (54.9-61.4)	1056/896	62.2 (58.0-66.4)	37.8 (33.6-42.0)	20.9 (17.4-24.4)	79.1 (75.6-82.6)	614/521
P-value		0.197			< 0.001		0.012	
**Education level**								
High-school degree or higher	43.4 (39.5-47.3)	56.6 (52.7-60.5)	1017/639	49.6 (44.4-54.8)	50.4 (45.2-55.6)	15.2 (11.5-18.9)	84.8 (81.1-88.5)	576/366
Less than high-school degree	43.8 (41.0-46.7)	56.2 (53.3-59.0)	1721/1214	55.5 (51.7-59.3)	44.5 (40.7-48.3)	18.6 (15.6-21.6)	81.4 (78.4-84.4)	967/683
P-value		0.858			0.072		0.166	
**Marital status**								
Married/engaged	38.7 (36.3-41.1)	61.3 (58.9-63.7)	2441/1663	52.9 (49.8-56.0)	47.1 (44.0-50.2)	17.8 (15.4-20.1)	82.2 (79.9-84.6)	1497/1022
Single/divorced/ widowed	84.6 (79.3-90.0)	15.4 (10.0-20.7)	297/190	65.3 (47.2-83.5)	34.7 (16.5-52.8)	3.9 (0.0-11.3)	96.1 (88.7-103.6)	46/27
P-value		< 0.001			0.207		0.066	
**Employment status**								

Employed	41.8 (39.2-44.5)	58.2 (55.5-60.8)	2111/1405	53.6 (50.1-57.1)	46.4 (42.9-49.9)	17.6 (15.0-20.2)	82.4 (79.8-85.0)	1228/822
Unemployed	49.9 (45.1-54.6)	50.1 (45.4-54.9)	626/448	52.1 (45.4-58.7)	47.9 (41.3-54.6)	16.3 (11.5-21.1)	83.7 (78.9-88.5)	314/227
P-value		0.003			0.694		0.649	
**Income level**								
High income (≥median)	43.8 (40.6-47.0)	56.2 (53.0-59.4)	1451/959	51.6 (47.3-55.9)	48.4 (44.1-52.7)	15.6 (12.6-18.7)	84.4 (81.3-87.4)	816/542
Low income (<median)	40.4 (36.3-44.4)	59.6 (55.6-63.7)	843/593	57.0 (51.7-62.2)	43.0 (37.8-48.3)	21.2 (16.9-25.5)	78.8 (74.5-83.1)	502/355
Not reported	49.6 (43.8-55.3)	50.4 (44.7-56.2)	444/301	51.1 (43.0-59.2)	48.9 (40.8-57.0)	14.9 (9.2-20.7)	85.1 (79.3-90.8)	223/152
P-value		0.037			0.256		0.076	
**Received cash assistance**								
No	45.7 (43.1-48.3)	54.3 (51.7-56.9)	2232/1451	51.3 (47.7-54.8)	48.7 (45.2-52.3)	16.0 (13.4-18.6)	84.0 (81.4-86.6)	1212/785
Yes	34.8 (30.1-39.6)	65.2 (60.4-69.9)	506/402	60.6 (54.6-66.5)	39.4 (33.5-45.4)	22.2 (17.1-27.2)	77.8 (72.8-82.9)	330/264
P-value		< 0.001			0.010		0.024	
**Self-rated physical health**								
Very good/ excellent	45.4 (40.1-50.8)	54.6 (49.2-59.9)	536/348	53.0 (45.8-60.3)	47.0 (39.7-54.2)	17.1 (11.6-22.5)	82.9 (77.5-88.4)	293/188
Good	45.8 (42.1-49.5)	54.2 (50.5-57.9)	1079/727	52.2 (47.2-57.2)	47.8 (42.8-52.8)	16.2 (12.6-19.8)	83.8 (80.2-87.4)	585/398
Poor/fair	40.7 (37.2-44.3)	59.3 (55.7-62.8)	1124/778	54.3 (49.7-59.0)	45.7 (41.0-50.3)	18.5 (14.9-22.0)	81.5 (78.0-85.1)	666/463
P-value		0.119			0.832		0.687	
**Anxiety severity**								
Minimal	43.9 (40.4-47.5)	56.1 (52.5-59.6)	1195/784	50.6 (45.9-55.3)	49.4 (44.7-54.1)	14.6 (11.4-17.9)	85.4 (82.1-88.6)	670/443
Mild	45.5 (41.3-49.8)	54.5 (50.2-58.7)	798/551	52.7 (47.0-58.5)	47.3 (41.5-53.0)	20.0 (15.4-24.6)	80.0 (75.4-84.6)	435/301
Moderate to severe	41.3 (36.9-45.6)	58.7 (54.4-63.1)	745/518	57.9 (52.2-63.6)	42.1 (36.4-47.8)	18.8 (14.4-23.2)	81.2 (76.8-85.6)	437/305
P-value		0.386			0.154		0.125	
**Depression severity**								
None to minimal	45.4 (41.0-49.7)	54.6 (50.3-59.0)	809/524	45.2 (39.4-51.1)	54.8 (48.9-60.6)	13.4 (9.4-17.4)	86.6 (82.6-90.6)	441/288
Mild	43.9 (39.9-47.9)	56.1 (52.1-60.1)	902/605	57.2 (51.8-62.5)	42.8 (37.5-48.2)	18.6 (14.4-22.8)	81.4 (77.2-85.6)	506/339
Moderate to severe	42.1 (38.4-45.8)	57.9 (54.2-61.6)	1028/724	55.9 (51.1-60.8)	44.1 (39.2-48.9)	19.2 (15.4-23.0)	80.8 (77.0-84.6)	595/422

P-value		0.527			0.005		0.110	
**Any chronic medical condition**								
No	44.5 (41.7-47.3)	55.5 (52.7-58.3)	1833/1248	52.2 (48.4-56.0)	47.8 (44.0-51.6)	17.8 (15.0-20.7)	82.2 (79.3-85.0)	1018/694
Yes	42.0 (38.0-46.0)	58.0 (54.0-62.0)	905/605	55.4 (50.1-60.7)	44.6 (39.3-49.9)	16.4 (12.5-20.2)	83.6 (79.8-87.5)	525/355
P-value		0.321			0.333		0.545	
**Any disability**								
No	44.1 (41.7-46.5)	55.9 (53.5-58.3)	2551/1718	52.5 (49.2-55.7)	47.5 (44.3-50.8)	17.5 (15.1-20.0)	82.5 (80.0-84.9)	1427/963
Yes	38.1 (29.7-46.6)	61.9 (53.4-70.3)	186/135	63.3 (52.9-73.7)	36.7 (26.3-47.1)	14.8 (7.0-22.7)	85.2 (77.3-93.0)	115/86
P-value		0.194			0.060		0.538	
**Water insecure**								
No	44.7 (41.7-47.7)	55.3 (52.3-58.3)	1630/1081	50.8 (46.7-54.8)	49.2 (45.2-53.3)	16.5 (13.5-19.5)	83.5 (80.5-86.5)	902/602
Yes	42.2 (38.6-45.7)	57.8 (54.3-61.4)	1108/772	56.8 (52.1-61.5)	43.2 (38.5-47.9)	18.5 (14.9-22.2)	81.5 (77.8-85.1)	641/447
P-value		0.288			0.058		0.388	
**Food insecure**								
No	53.0 (47.7-58.3)	47.0 (41.7-52.3)	573/350	49.2 (41.5-57.0)	50.8 (43.0-58.5)	12.2 (7.2-17.2)	87.8 (82.8-92.8)	270/164
Yes	41.2 (38.6-43.7)	58.8 (56.3-61.4)	2164/1503	54.1 (50.8-57.5)	45.9 (42.5-49.2)	18.4 (15.8-21.0)	81.6 (79.0-84.2)	1273/885
P-value		< 0.001			0.256		0.053	

CI – confidence interval, LARC – long-acting reversible contraception

*Reported p-values were extracted from χ^2 tests, assessing contraceptive use differences among subgroups of the population.

Table S5: Weighted prevalence estimates (in %) of unmet need for family planning and demand satisfied with modern methods among men aged 19-64 years in Lebanon in 2024, overall and stratified by social determinant

Parameter	Prevalence of unmet need for family planning (95% CI)*	Percentage of demand satisfied with modern methods (95% CI)*	Denominator (weighted/ unweighted)
**Total**	36.4 (34.0-38.8)	33.9 (31.5-36.2)	2343/1576
**Age in years**			
19–29	56.4 (50.4-62.4)	26.3 (21.0-31.5)	387/273
30–39	30.8 (26.4-35.1)	38.5 (34.0-43.0)	657/460
40–49	19.9 (16.2-23.6)	40.7 (36.1-45.3)	669/450
50-59	39.4 (33.7-45.0)	32.3 (26.9-37.8)	464/293
60-64	70.1 (61.1-79.1)	10.5 (4.6-16.5)	166/100
**Nationality**			
Lebanese	39.1 (35.8-42.4)	29.0 (25.9-32.1)	1474/839
Non-Lebanese	31.9 (28.5-35.2)	42.2 (38.7-45.8)	869/737
**Education level**			
High-school degree or higher	37.6 (33.5-41.7)	30.8 (27.0-34.6)	894/560
Less than high-school degree	35.7 (32.7-38.7)	35.8 (32.8-38.8)	1449/1016
**Marital status**			
Married/engaged	29.4 (27.0-31.9)	37.3 (34.7-39.9)	2047/1387
Single/divorced/widowed	84.6 (79.2-89.9)	10.1 (5.6-14.5)	296/189
**Employment status**			
Employed	34.2 (31.5-37.0)	35.4 (32.6-38.1)	1799/1191
Unemployed	43.5 (38.4-48.6)	29.0 (24.4-33.6)	544/385
**Income level**			
High income (≥median)	36.8 (33.4-40.2)	32.8 (29.5-36.0)	1242/817
Low income (<median)	30.3 (26.1-34.5)	39.5 (35.1-43.9)	698/488
Not reported	45.7 (39.7-51.8)	27.5 (22.1-32.9)	403/271
**Received cash assistance**			
No	38.4 (35.6-41.1)	31.6 (29.0-34.3)	1908/1232
Yes	27.8 (22.9-32.7)	43.7 (38.4-49.0)	435/344
**Self-rated physical health**			
Very good/excellent	36.1 (30.5-41.7)	34.0 (28.5-39.6)	449/290
Good	38.7 (34.7-42.6)	32.6 (28.8-36.3)	912/612
Poor/fair	34.4 (30.7-38.1)	35.0 (31.4-38.7)	982/674
**Anxiety severity**			
Minimal	37.0 (33.2-40.7)	32.3 (28.7-35.9)	1022/666
Mild	37.4 (32.9-42.0)	32.3 (28.0-36.6)	668/459
Moderate to severe	34.5 (30.0-39.0)	38.0 (33.4-42.5)	653/451
**Depression severity**			
None to minimal	38.6 (34.0-43.2)	28.1 (23.9-32.3)	697/450

Mild	37.0 (32.8-41.3)	36.0 (31.8-40.2)	769/512
Moderate to severe	34.1 (30.3-37.9)	36.6 (32.8-40.5)	877/614
**Any chronic medical condition**			
No	36.1 (33.1-39.1)	33.5 (30.6-36.4)	1527/1033
Yes	37.0 (32.8-41.2)	34.7 (30.6-38.7)	816/543
**Any disability**			
No	37.0 (34.4-39.5)	33.1 (30.6-35.5)	2183/1460
Yes	28.6 (20.1-37.2)	44.9 (35.7-54.1)	160/116
**Water insecure**			
No	38.3 (35.1-41.5)	31.4 (28.4-34.4)	1415/932
Yes	33.5 (29.8-37.3)	37.7 (33.9-41.5)	928/644
**Food insecure**			
No	48.7 (43.0-54.4)	26.1 (21.1-31.1)	495/302
Yes	33.1 (30.5-35.8)	36.0 (33.3-38.7)	1848/1274

CI – confidence interval.

*Definitions of the indicators follow those provided by the United Nations Population Fund State of the World Population Report.

Table S6: Distribution of contraceptive methods reported by men aged 19-64 years with no pregnancy intentions in Lebanon in 2024, including methods used by themselves or their partners, stratified by marital status*

Method of Contraception	Total (n = 1576)	Married/engaged (n = 1387)	Single/divorced/ widowed (n = 189)
**Nothing**	563 (36.4%)	401 (29.5%)	162 (84.5%)
**Natural methods**			
Withdrawal†	441 (28.5%)	432 (31.9%)	9 (5.4%)
Periodic abstinence†	32 (2.0%)	32 (2.3%)	0 (0.0%)
LAM‡	7 (0.5%)	7 (0.5%)	0 (0.0%)
**Modern methods**			
LARC methods			
Intrauterine device‡	178 (10.8%)	177 (12.3%)	1 (0.7%)
Contraceptive injection‡	3 (0.2%)	3 (0.2%)	0 (0.0%)
Contraceptive implant‡	1 (0%)	1 (0%)	0 (0.0%)
Non-LARC modern methods			
Oral contraceptive pill‡	208 (12.0%)	206 (13.6%)	2 (0.7%)
Male condom§	137 (9.0%)	122 (9.1%)	15 (8.8%)
Female sterilisation‡	32 (2.1%)	32 (2.4%)	0 (0.0%)
Female condom‡	5 (0.3%)	5 (0.4%)	0 (0.0%)
Diaphragm‡	2 (0.1%)	2 (0.1%)	0 (0.0%)
Male sterilisation§	1 (0.1%)	1 (0.1%)	0 (0.0%)
Contraceptive gels‡	0 (0.0%)	0 (0.0%)	0 (0.0%)

LAM – lactational amenorrhea method, LARC – long-acting reversible contraception
*Presented as n (weighted %).
†Applicable to males and females.
‡Female-specific.
§Male-specific.

Table S7: Adjusted odds ratio estimates and 95% confidence intervals from survey-weighted logistic regression models for the effects of each determinant on method of contraception used among women aged 19-49 years with no pregnancy intentions

	**Contraceptive use aOR (95% CI)**		
	Any vs None†	Modern vs Natural‡	LARC vs non-LARC§
**Age in years**			
19–29	1	1	1
30–39	2.24 (1.61-3.14)*	0.89 (0.59-1.34)	0.88 (0.53-1.44)
40–49	1.49 (1.06-2.09)*	0.99 (0.64-1.53)	0.81 (0.47-1.38)
**Nationality**			
Lebanese	1	1	1
Non-Lebanese	1.44 (1.08-1.91)*	1.39 (1.01-1.90)*	1.53 (1.04-2.25)*
**Education level**			
High-school degree or higher	1	1	1
Less than high-school degree	0.87 (0.65-1.17)	1.18 (0.85-1.64)	1.14 (0.74-1.75)
**Marital status**			
Married/engaged	1	1	1
Single/divorced/widowed	0.02 (0.01-0.05)*	2.41 (0.44-13.09)	-
**Employment status**			
Employed	1	1	1
Unemployed	0.96 (0.70-1.32)	0.96 (0.70-1.31)	0.76 (0.52-1.12)
**Income level**			
High income (≥median)	1	1	1
Low income (<median)	1.07 (0.67-1.70)	0.89 (0.56-1.40)	0.76 (0.43-1.32)
Not reported	1.09 (0.66-1.80)	0.79 (0.48-1.30)	1.08 (0.57-2.03)
**Received cash assistance**			
No	1	1	1
Yes	1.11 (0.76-1.62)	1.33 (0.88-2.00)	1.45 (0.94-2.25)
**Self-rated physical health**			
Very good/excellent	1	1	1
Good	1.23 (0.74-2.02)	1.13 (0.62-2.05)	0.82 (0.37-1.80)
Poor/fair	1.66 (1.01-2.77)*	0.95 (0.52-1.73)	0.93 (0.42-2.06)
**Anxiety severity**			
Minimal	1	1	1
Mild	1.14 (0.81-1.62)	0.99 (0.66-1.49)	0.81 (0.49-1.36)
Moderate to severe	1.33 (0.92-1.92)	1.30 (0.84-2.00)	0.94 (0.55-1.59)
**Depression severity**			
None to minimal	1	1	1
Mild	0.92 (0.63-1.37)	1.20 (0.75-1.92)	1.55 (0.85-2.84)

Moderate to severe	1.12 (0.75-1.67)	1.08 (0.68-1.72)	1.14 (0.63-2.08)
**Any chronic medical condition**			
No	1	1	1
Yes	0.95 (0.71-1.27)	1.37 (0.98-1.91)	1.10 (0.72-1.66)
**Any disability**			
No	1	1	1
Yes	1.39 (0.66-2.92)	0.79 (0.38-1.65)	1.80 (0.80-4.04)
**Water insecure**			
No	1	1	1
Yes	1.18 (0.86-1.62)	0.91 (0.66-1.24)	0.74 (0.50-1.09)
**Food insecure**			
No	1	1	1
Yes	0.95 (0.60-1.52)	0.90 (0.57-1.42)	0.51 (0.29-0.87)*

Confounders for each model can be found in Table S1
aOR – adjusted odds ratio, CI – confidence interval
*p-value < 0.05
†Any = 1; None = 0
‡Modern = 1; Natural = 0
§LARC = 1; Other = 0

Table S8: Adjusted odds ratio estimates and 95% confidence intervals from survey-weighted logistic regression models for the effects of each determinant on method of contraception used among men aged 19-64 years with no pregnancy intentions

	**Contraceptive use aOR (95% CI)**		
	Any vs None†	Modern vs Natural‡	LARC vs non-LARC§
**Age in years**			
19-29	1	1	1
30-39	2.91 (2.12-4.00)*	0.82 (0.53-1.27)	0.65 (0.39-1.09)
40-49	5.22 (3.72-7.32)*	0.68 (0.44-1.04)	0.68 (0.41-1.13)
50-59	1.99 (1.42-2.80)*	0.75 (0.47-1.21)	0.67 (0.37-1.19)
60-64	0.55 (0.34-0.91)*	0.36 (0.16-0.83)*	0.34 (0.09-1.23)
**Nationality**			
Lebanese	1	1	1
Non-Lebanese	1.30 (1.04-1.64)*	1.73 (1.34-2.25)*	1.49 (1.07-2.08)*
**Education level**			
High-school degree or higher	1	1	1
Less than high-school degree	0.95 (0.75-1.20)	1.11 (0.84-1.47)	1.19 (0.81-1.74)
**Marital status**			
Married/engaged	1	1	1
Single/divorced/widowed	0.08 (0.05-0.12)	1.55 (0.68-3.53)	0.16 (0.02-1.24)
**Employment status**			
Employed	1	1	1
Unemployed	0.69 (0.52-0.92)*	0.81 (0.59-1.12)	0.88 (0.58-1.33)
**Income level**			
High income (≥median)	1	1	1
Low income (<median)	1.10 (0.83-1.45)	1.13 (0.84-1.51)	1.35 (0.94-1.94)
Not reported	0.71 (0.50-1.01)	0.96 (0.65-1.43)	0.92 (0.53-1.59)
**Received cash assistance**			
No	1	1	1
Yes	1.29 (0.94-1.76)	1.08 (0.77-1.51)	1.35 (0.90-2.03)
**Self-rated physical health**			
Very good/excellent	1	1	1
Good	0.79 (0.57-1.09)	0.88 (0.60-1.28)	0.89 (0.55-1.46)
Poor/fair	0.85 (0.60-1.21)	0.79 (0.53-1.18)	0.93 (0.55-1.58)
**Anxiety severity**			
Minimal	1	1	1
Mild	0.81 (0.61-1.08)	0.94 (0.68-1.30)	1.32 (0.87-2.00)
Moderate to severe	0.88 (0.66-1.17)	1.15 (0.83-1.60)	1.18 (0.75-1.84)
**Depression severity**			

None to minimal	1	1	1
Mild	0.93 (0.69-1.25)	1.56 (1.11-2.21)*	1.37 (0.84-2.21)
Moderate to severe	0.96 (0.71-1.30)	1.30 (0.92-1.84)	1.28 (0.79-2.09)
**Any chronic medical condition**			
No	1	1	1
Yes	1.00 (0.78-1.28)	1.24 (0.94-1.64)	0.95 (0.66-1.39)
**Any disability**			
No	1	1	1
Yes	1.43 (0.90-2.27)	1.54 (0.97-2.46)	0.81 (0.42-1.56)
**Water insecure**			
No	1	1	1
Yes	0.98 (0.77-1.25)	1.21 (0.93-1.57)	1.11 (0.79-1.56)
**Food insecure**			
No	1	1	1
Yes	1.09 (0.79-1.52)	0.90 (0.62-1.31)	1.34 (0.78-2.30)

Confounders for each model can be found in Table S1
aOR – adjusted odds ratio, CI – confidence interval
*p-value < 0.05
†Any = 1; None = 0
‡Modern = 1; Natural = 0
§LARC = 1; Other = 0

Figure S1: Forest plot for the adjusted odds ratio estimates and 95% confidence intervals from survey-weighted logistic regression models for the effects of each determinant on method of contraception used among men with no pregnancy intentions

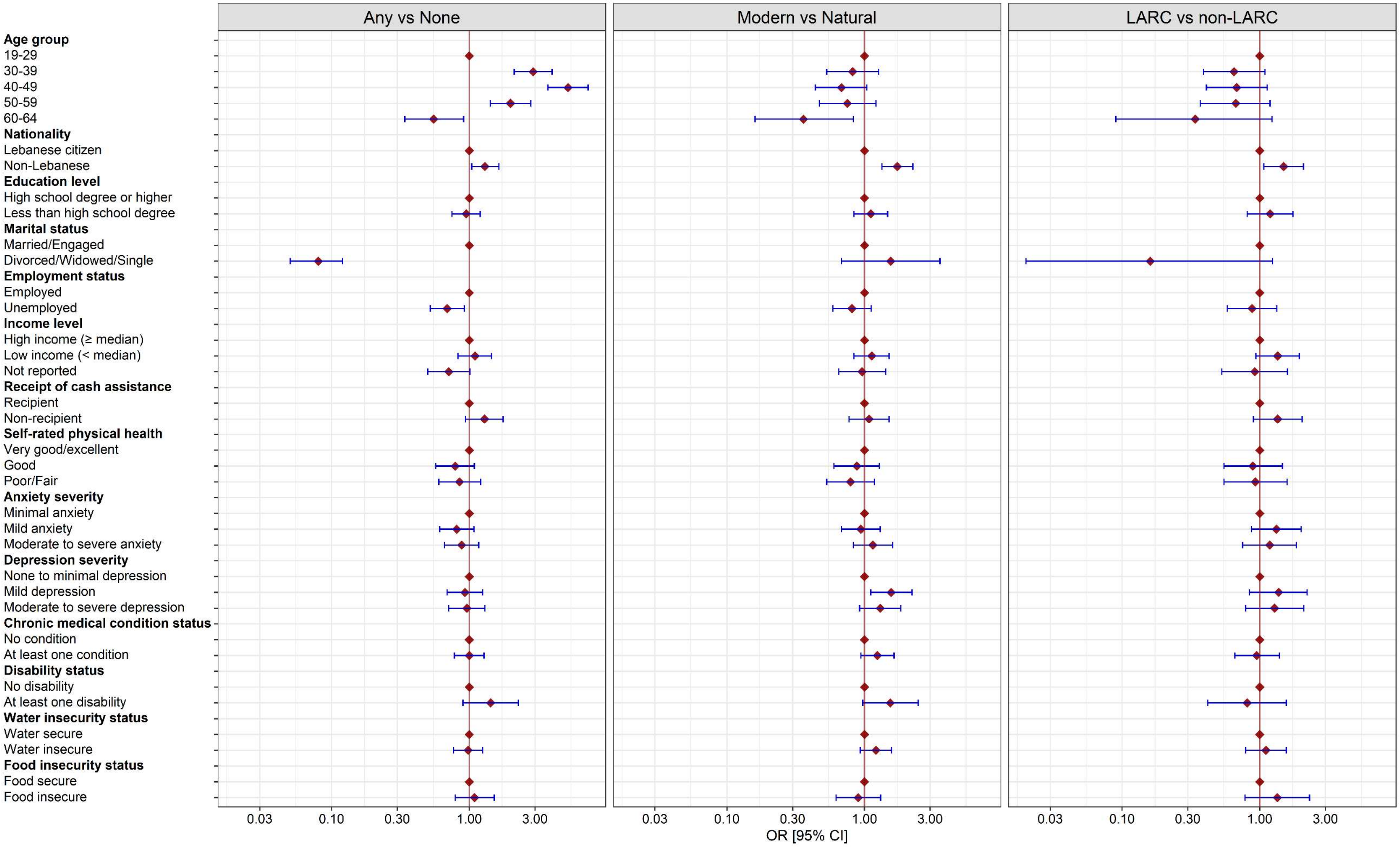

Confounders for each model can be found in Table S1. Outcome coding: Any = 1, None = 0; Modern = 1, Natural = 0; LARC = 1, non-LARC = 0. CI – confidence interval, OR – odds ratio.
